# Cisd2 Protects the Liver from Oxidative Stress and Ameliorates Western Diet-Induced Nonalcoholic Fatty Liver Disease

**DOI:** 10.3390/antiox10040559

**Published:** 2021-04-03

**Authors:** Yi-Long Huang, Zhao-Qing Shen, Chen-Hua Huang, Yuan-Chi Teng, Chao-Hsiung Lin, Ting-Fen Tsai

**Affiliations:** 1 Department of Life Sciences and Institute of Genome Sciences, National Yang Ming Chiao Tung University, Taipei 112, Taiwan; yilonghuang@nycu.edu.tw (Y.-L.H.); d40008003@ym.edu.tw (Z.-Q.S.); denny1210.y@nycu.edu.tw (C.-H.H.); andrea_chi@ym.edu.tw (Y.-C.T.); 2 Aging and Health Research Center, National Yang Ming Chiao Tung University, Taipei 112, Taiwan; 3 Institute of Molecular and Genomic Medicine, National Health Research Institutes, Zhunan 350, Taiwan; 4 Institute of Biotechnology and Pharmaceutical Research, National Health Research Institutes, Zhunan 350, Taiwan

**Keywords:** nonalcoholic fatty liver disease (NAFLD), nonalcoholic steatohepatitis (NASH), Cisd2, Western diet, transcriptomics, hepatocyte-specific knockout

## Abstract

Nonalcoholic fatty liver disease (NAFLD) and its more severe form, nonalcoholic steatohepatitis (NASH), are the most common chronic liver diseases worldwide. However, drugs to treat NAFLD and NASH are an unmet clinical need. This study sought to provide evidence that Cisd2 is a molecular target for the development of treatments targeting NAFLD and NASH. Several discoveries are pinpointed. The first is that Cisd2 dosage modulates the severity of Western diet-induced (WD-induced) NAFLD. Specifically, Cisd2 haploinsufficiency accelerates NAFLD development and exacerbates progression toward NASH. Conversely, an enhanced Cisd2 copy number attenuates liver pathogenesis. Secondly, when a WD is fed to mice, transcriptomic analysis reveals that the major alterations affecting biological processes are related to inflammation, lipid metabolism, and DNA replication/repair. Thirdly, among these differentially expressed genes, the most significant changes involve Nrf2-mediated oxidative stress, cholesterol biosynthesis, and fatty acid metabolism. Finally, increased Cisd2 expression protects the liver from oxidative stress and reduces the occurrence of mitochondrial DNA deletions. Taken together, our mouse model reveals that Cisd2 plays a crucial role in protecting the liver from WD-induced damages. The development of therapeutic agents that effectively enhance Cisd2 expression is one potential approach to the treatment of WD-induced fatty liver diseases.

## 1. Introduction

Nonalcoholic fatty liver disease (NAFLD) is a continuous spectrum of diseases characterized by excessive fat accumulation in the hepatocytes of the liver. NAFLD, which affects approximately 25% of the global population, is the most common liver disorder worldwide [1]. In the early stage of NAFLD, simple hepatic steatosis is not very dangerous and usually is reversible. Risk factors for this disease include diabetes, obesity, diets with high content of either fat or fructose, and an older age. Specifically, for individuals with obesity-induced NAFLD, weight loss, through either exercise or diet, is the most effective way of reducing liver fat and bring about a recovery in liver function. Importantly, a significant portion of NAFLD may progress to its more severe form, which is nonalcoholic steatohepatitis (NASH); the latter disease is characterized by the presence of inflammation and fibrosis. NASH usually leads to various complications, including cirrhosis, hepatocellular carcinoma (HCC), liver failure, and/or cardiovascular disease. However, at present, no drugs that specifically target NAFLD or NASH have received approval [2,3]. Accordingly, medication for treating NAFLD and NASH remains an unmet clinical need and new interventions and novel therapeutic approaches are urgently needed.

The synergistic effects of multiple factors, including insulin resistance, dysregulation of lipid metabolism, chronic inflammation, and oxidative stress, are all involved in the occurrence of NAFLD and its progression to NASH [3,4]. Currently, an increasing number of studies have suggested that reactive oxygen species (ROS) and/or reactive nitrogen species (RNS) seem to play a crucial role in the progression of the liver through the various pathogenic stages that lead toward NAFLD and NASH [5,6]. In addition, previous studies have revealed that an increased level of ROS results in lipid peroxidation, protein oxidation and inflammation; these can form a vicious cycle and result in various types of hepatocyte cell death, which in turn brings about chronic liver damage [7]. However, it is not completely understood how ROS and/or RNS trigger inflammation and fibrogenesis, thus aggravating the development of this type of liver pathology.

Cisd2 is a pro-longevity gene that mediates lifespan in mammals. Cisd2 deficiency causes a premature aging phenotype in Cisd2 knockout (Cisd2KO) mice. Conversely, a persistently high level of Cisd2 delays aging and mitigates age-related functional decline in multiple tissues, including skeletal muscle, neurons, skin, and heart [8,9,10,11,12,13]. In addition, Cisd2 has been detected to be associated with mitochondrial outer membranes, the ER, and mitochondria-associated ER membranes (MAMs) in various cell types. ER and mitochondria are the two major intracellular Ca^2+^ stores that respond to the signals for Ca^2+^ mobilization, while MAMs serve as hotspots for Ca^2+^ transfer between the ER and mitochondria. Studies from our group and other laboratories have revealed that Cisd2 plays an essential role in mitochondrial integrity and intracellular Ca^2+^ homeostasis [11,14,15,16,17,18,19,20]. Furthermore, Cisd2 deficiency leads to mitochondrial dysfunction and structural breakdown [13]. Taken together, these findings suggest that Cisd2 is a fundamentally important regulator of lifespan and metabolism via the maintenance of intracellular Ca^2+^ homeostasis and mitochondrial integrity.

In the liver, one of our previous studies revealed that Cisd2 haploinsufficiency predisposes mice to the development of NAFLD and NASH with a 100% penetrance; this was found to occur in heterozygous mice carrying a hepatocyte-specific Cisd2KO (Cisd2hKO^+/−^). Remarkably, at the young age of 3-month-old, Cisd2hKO^+/−^ mice can be seen to have developed hepatic steatosis, hepatic inflammation, hepatic cell death, hepatic fibrosis, and chronic liver regeneration. The magnitude of these pathological changes is exacerbated as the mice get older. On reaching middle age, or 12-months old, 100% of the Cisd2hKO^+/−^ mice have severe NASH, and about 20% of these mice spontaneously develop HCC before 24-months old. Furthermore, Cisd2 haploinsufficiency is a risk factor that also accelerates HBV-associated and DEN-induced HCC [20,21]. On the other hand, using Cisd2 transgenic (Cisd2TG) mice, the increased Cisd2 expression is able to suppress HBV-associated and DEN-induced HCC. These results demonstrate that Cisd2 is a haploinsufficient tumor-suppressor gene in the mouse liver and that increased expression of Cisd2 is able to attenuate hepatocarcinogenesis and suppress HCC development [20,21].

The Western diet is a modern dietary pattern that is generally characterized by high intakes of red meat, butter, fried foods, high-fat dairy products, refined grains, high-fructose corn syrup, artificially sweetened foods and high-sugar drinks. By way of contrast, a healthy diet that also helps to protect against malnutrition is usually considered to emphasize a higher proportion in the diet of white meat, fish, whole-grain foods, fat-free or low-fat foods, unprocessed fruits, nuts, vegetables, and legumes. The Western diet has been positively associated with an increased incidence of obesity and various obesity-related diseases, with the latter including metabolic syndrome and a number of cardiovascular diseases [22]. In particular, a Western diet is considered to be one of the major risk factors for NAFLD and NASH [3]. The composition of a diet in terms of nutrients is well known to have a profound impact on the NAFLD disease progression. A number of Western diet foods have been used to simulate various features of human NAFLD in wild type or genetically modified mice [23,24,25]. However, only a few studies have examined the global transcriptomic changes in the liver when an experimental subject is being fed a Western diet for a specific period. Therefore, the remodeling of the liver’s metabolic pathways, as well as the molecular alterations that take place in the liver linked to the lipid accumulation together with the pro-inflammatory response associated with liver pathogenesis, remain elusive.

Our success in discovering that Cisd2 plays an essential role in the liver has motivated our efforts to explore whether an enhanced level of Cisd2 has beneficial effects when mice have NAFLD or NASH that have been induced by a Western diet. Furthermore, using transcriptomics, our aim was to obtain insights into the molecular mechanism(s) underlying the dose-dependent effects of Cisd2 on the associated liver pathogenesis; specifically, the detrimental effects on liver that Cisd2 haploinsufficiency brings about in heterozygous mice with a hepatocyte-specific Cisd2hKO^+/−^ background. We also wanted to explore further the protective effects on the liver brought by an approximately two-fold overexpression of Cisd2 in the Cisd2TG mice, which is our long-lived mouse model. Our mouse studies should provide experimental evidence that will form the basis of a paradigm regarding the functioning of Cisd2 in the development of NAFLD and NASH, and should also help the development of therapeutic strategies aimed at treating NAFLD and NASH.

## 2. Materials and Methods

### 2.1. Mice

The Cisd2 BAC transgenic (Cisd2TG) mice [20] and the Cisd2 floxed allele (Cisd2^f/f^) mice [17] were generated as previously described. To generate mice with a heterozygous hepatocyte-specific Cisd2 knockout (Cisd2hKO^+/−^, Cisd2^f/+^; Alb-Cre) in their livers, the Cisd2^f/f^ mice were bred with Albumin-Cre transgenic (Alb-Cre, JAX003574) mice. Male C57BL/6 mice were employed for all experiments. The mice were maintained at 21 ± 1 °C with a 12 h light/dark cycle and had free access to food and water under specific pathogen-free conditions. For the diet-induced fatty liver model, a high fat and high sucrose Western-style diet (Research Diet D12079B) was fed to the WT, Cisd2TG, and Cisd2hKO^+/−^ mice from 2 months of age until 6 months of age. The body weights and liver weights of the mice were recorded after the mice had been euthanized. The serum concentration of alanine aminotransferase (ALT) of each mouse was determined by DRI-CHEM 3500 s (FUJIFILM). All animal protocols were approved by the Institutional Animal Care and Use Committee (IACUC) of National Yang Ming Chiao Tung University (No. 1080410). The animal study was designed to comply with the associated guidelines and 3R (Replacement, Reduction, and Refinement) principle in accordance with the law of “Animal Protection Act” in Taiwan.

### 2.2. Liver Histopathology

Liver tissue samples were collected, then fixed in 10% buffered formalin solution, before undergoing tissue processing and paraffin embedding. Hematoxylin & Eosin (H&E) and Masson’s trichrome staining (Muto Pure Chemicals, Tokyo, Japan) were carried out on liver sections (3 μm) and were conducted using their standard protocols.

### 2.3. Immunohistochemistry

Immunohistochemistry (IHC) staining was carried out on paraffin-embedded liver sections (3 μm) using anti-F4/80 (123101, BioLegend, San Diego, USA) antibody, which was followed by counterstaining with hematoxylin. To do this, sections were deparaffinized, rehydrated, and antigen-retrieved using a target retrieval solution (S1699, Dako, Glostrup, Denmark). Subsequently, the liver sections were quenched using 3% H2O2 in PBS, blocked using 5% bovine serum albumin in PBS, incubated with the primary antibody in antibody diluent (ab64211, Abcam, Cambridge, UK) and finally detected using a LSAB kit (K0679, Dako, Glostrup, Denmark).

### 2.4. Western Blotting

Liver tissue samples were homogenized in lysis buffer containing 50 mM Tris at pH 7.4, 100 mM NaCl, 1 mM EDTA, and 1% Triton X-100, as well as in the presence of complete protease inhibitor cocktail and phosphatase inhibitor cocktail (Roche, Basel, Switzerland). Samples containing a total of 10 μg tissue homogenate protein per lane were separated by 12% SDS-PAGE and the separated proteins were then transferred onto polyvinylidene fluoride (PVDF) membranes. Each membrane was blocked using 5% (*w*/*v*) nonfat dry milk, probed with Cisd2 [8] or Gapdh (MAB374, Millipore, Burlington, MA, USA) antibody, and finally developed using a ECL reagent kit (34580, Thermo Fischer Scientific, Waltham, MA, USA).

### 2.5. Measurement of Hepatic Triglyceride, MDA, and ROS/RNS Levels

Liver triglycerides (TriGs) ere extracted by homogenization of the liver tissue samples with a mixture of 1:2 chloroform and methanol (*v*/*v*) as described previously [26]. The hepatic triglyceride content of each sample was determined using a triglyceride assay kit (TR0100, Sigma-Aldrich, Munich, Germany). Hepatic levels of the lipid peroxidation product malondialdehyde (MDA) were determined using thiobarbituric acid reactive substances (TBARS) via a colorimetric kit (STA-330, Cell Biolabs, San Diego, CA, USA). Quantitative determination of liver ROS/RNS was carried out in vitro using a ROS/RNS assay kit (STA-347, Cell Biolabs).

### 2.6. Mitochondrial DNA Deletion

The presence of deletions of the mitochondria’s DNA was identified as previously described using diagnostic PCR fragments amplified from genomic DNA isolated from livers tissue samples [27].

### 2.7. RNA Extraction, Sequencing, and Analysis

Total RNA was isolated from mouse liver tissues using TRI Reagent (T9424, Sigma) and phenol/chloroform extraction. RNA sequencing was performed by National Yang-Ming University VYM Genome Research Center. The analysis was generated to a depth of at least 20 million reads for each sample by single-end sequencing. After mapping, the unique gene reads were analyzed as TPM (transcripts per million) to identify differential expression. A total of 10,803 genes were retained after filtering to identify expressed genes (minimal counts in TPMs > 1 as detected in at least 50% of samples) using a two-tailed Student’s *t*-test. The genes, with *p* values, have been adjusted using the Benjamini–Hochberg method. Differentially expressed genes (DEGs) were identified using a cut-off threshold of false discovery rate (FDR), and the fold-change for each gene is indicated in each figure. Gene ontology annotations of the DEGs were carried out using the statistical overrepresentation test of PANTHER (http://pantherdb.org/, accessed on 15 November 2020) using the GO-Slim Biological Process. Significance was set at a fold enrichment >4 and an FDR of <5% for the GOslim analysis. Upstream regulator analysis and Ingenuity Tox List tools were used on the identified genes and were performed using Ingenuity Pathways Analysis (IPA; http://www.ingenuity.com, accessed on 25 November 2020). The top relevant ontologies that were able to give biological insights in liver metabolism are presented.

### 2.8. Statistical Analysis

The data are presented as mean ± SD as described in the figure legends. Comparisons between two groups were carried out using an unpaired two-tailed Student’s *t*-test. Significant differences were defined as *p* < 0.05. The RNA-seq data in TPM were loaded into the EZinfo 3.0.3 software (Umetrics, Umeå, Sweden) to allow principal component analysis (PCA). The TPM values were transformed into z-scores to allow presentation as heatmaps, and these were generated by the Multi Experiment Viewer (MEV) 4.9 software [28].

## 3. Results

### 3.1. Cisd2 Modulates Western Diet-Induced NAFLD and NASH in a Dose-Dependent Manner

**Figure 1:**
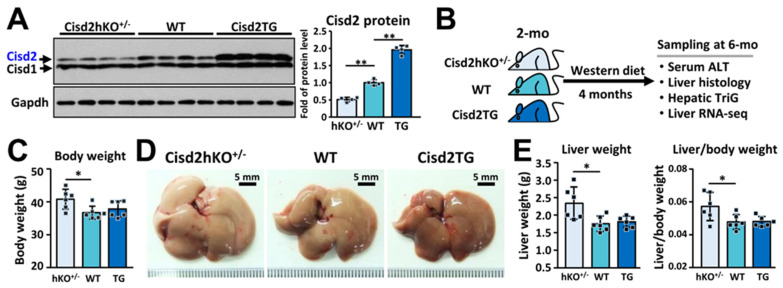
Cisd2 is a critical determinant in Western diet (WD)-induced fatty liver. (**A**) Immunoblots of Cisd2 protein in the livers of Cisd2hKO^+/−^, WT, and Cisd2TG mice (*n* = 4). (**B**) Sampling schedule of the transcriptome profiling of mice. Animals were fed ad libitum with WD from 2-month-old to 6-month-old. (**C**) Body weight after WD treatment for 4 months. (*n* = 6). (**D**) Gross view of liver after WD treatment for 4 months. (**E**) Liver weight and ratios of liver/body weight after WD treatment for 4 months. Mouse number *n* = 6. Data are presented as mean ± SD. * *p* < 0.05; ** *p* < 0.005.

A Western diet (WD), which contains 40% energy from fat, 43% energy from carbohydrate, and 0.15% cholesterol (*w*/*w*), has been developed that mimics the equivalent human Western diet [29]. To test whether the genetic dosage of Cisd2 modulates the severity of pathogenesis of WD-induced fatty liver, mice having different copy numbers of Cisd2 in their livers and thus expressing different levels of Cisd2 protein (Figure 1A), namely Cisd2hKO^+/−^ (50%), WT (100%), and Cisd2TG (200%), were fed the WD for 4 months. The mice were fed the WD starting at 2-month-old and were analyzed at 6-month-old (Figure 1B). Notably, Cisd2hKO^+/−^ mice showed a greater gain in body weight compared with the WT and Cisd2TG mice; however, there is no significant difference between the WT and Cisd2TG mice (Figure 1C). Interestingly, the morphology of the livers obtained from the WD-treated Cisd2hKO^+/−^ mice were lighter in color and looked bigger in size; indeed, in the Cisd2hKO^+/−^ mice, there was a significant increase in the weights of the livers, as well as an increase in the ratio of liver weight to body weight (Figure 1D,E).

**Figure 2:**
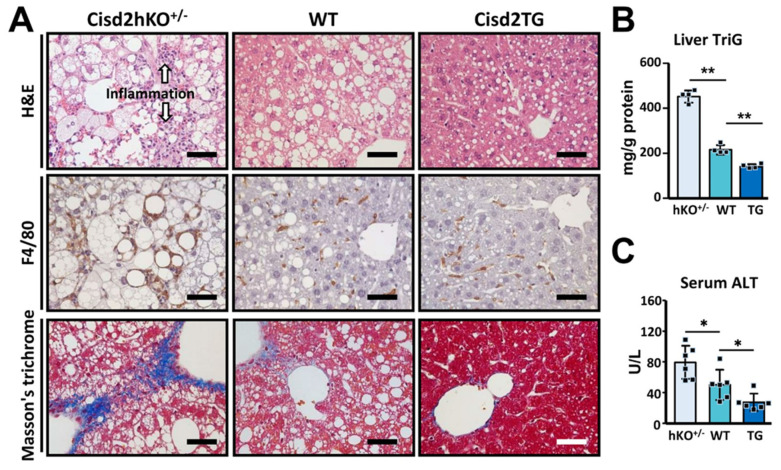
Cisd2 modulates WD-induced nonalcoholic fatty liver disease (NAFLD) and nonalcoholic steatohepatitis (NASH) in a dose-dependent manner. (**A**) Representative images of H&E, IHC staining of F4/80 (Kupffer cell marker) and Masson’s trichrome staining of liver sections prepared from WD-fed mice at 6-month-old. Hepatic steatosis, hepatocyte ballooning, and degeneration, as well as overt inflammation are detected in the livers of Cisd2hKO^+/−^ mice. (**B**) Liver triglyceride levels (*n* = 4). (**C**) Serum ALT levels (*n* = 6). Scale bar, 50 μm. Data are presented as mean ± SD. * *p* < 0.05; ** *p* < 0.005.

In terms of pathology, when the WT mice were examined, WD feeding was found to induce overt fat accumulation in the liver, as evidenced by H&E staining of liver sections (Figure 2A). Strikingly, in the WD-fed Cisd2hKO^+/−^ mice, there was a significant increase in fat accumulation and this was accompanied by inflammation, both being able to be observed in the H&E stained liver sections. Furthermore, IHC staining of F4/80 was used to validate the presence of inflammation and to locate the macrophages that had accumulated around the lipid droplets within the livers of the WD-fed Cisd2hKO^+/−^ mice. In addition, severe liver fibrosis was also detectable in the WD-fed Cisd2hKO^+/−^ mice, as indicated by Masson’s trichrome staining (Figure 2A). Remarkably, in the WD-fed Cisd2TG mice after 4 months, the two-fold overexpression of Cisd2 could be seen to have obviously brought about a decrease in lipid droplet deposition, while at the same time there had been a discernible attenuation of liver inflammation and reduced liver fibrosis (Figure 2A). Consistent with these pathological findings, a significant increase in the levels of hepatic triglyceride (TriG) and serum alanine aminotransferase (ALT), the latter being a liver damage marker, was found in the WD-fed Cisd2hKO^+/−^ mice compared with the WD-fed WT mice. By way of contrast, among the WD-fed Cisd2TG mice, the levels of hepatic TriG and serum ALT were significantly lower compared with the WD-fed WT controls (Figure 2B,C). Taken together, these findings reveal that the dosage of Cisd2 modulates the level of severity of WD-induced NAFLD. Cisd2 haploinsufficiency, namely a half dose of Cisd2, is insufficient, and this state accelerates WD-induced liver pathogenesis in the liver and exacerbates its progression toward NASH. Conversely, an enhanced level of Cisd2 is able to attenuate WD-induced NAFLD thereby preventing the development of NASH at a later stage.

### 3.2. Consistency between Transcriptomics Analysis and Liver Pathogenesis

**Figure 3:**
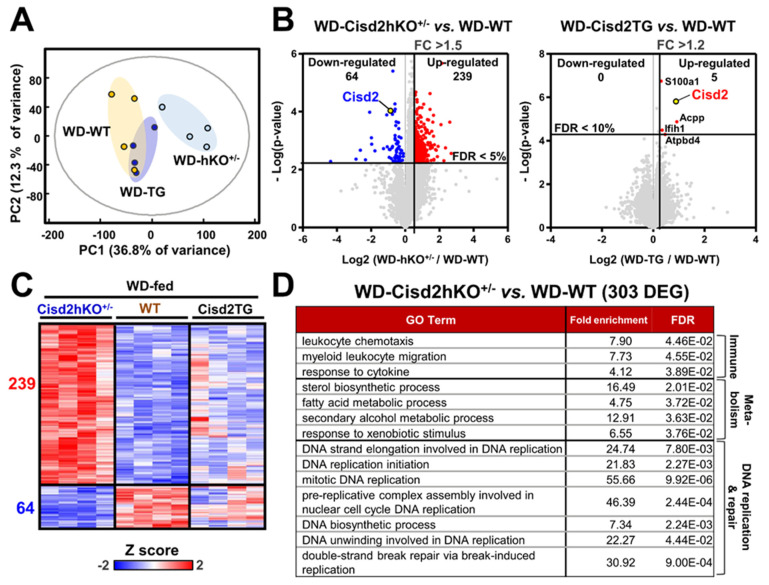
Cisd2 modulates hepatic gene expression profiles in WD-treated mice. (**A**) Principal component analysis (PCA) of all transcriptomic data for the 3 groups of mice (WD-fed WT, WD-fed Cisd2hKO^+/−^, and WD-fed Cisd2TG mice). (**B**) Volcano plots comparing mRNA expression between Cisd2hKO^+/−^ vs. WT (left panel) and between Cisd2TG vs. WT (right panel). (**C**) Heatmap representing the Cisd2hKO^+/−^-induced 303 DEGs. (**D**) Over-represented PANTHER GO-Slim Biological Processes of transcriptome change (303 DEGs). Top GO terms with fold enrichment above 4 are shown.

RNA-sequencing was used in this study to examine the molecular details and pathway changes that underlying the metabolic perturbations occurring during WD-induced NAFLD and NASH. Global transcriptomic profiling at the mRNA level was able to roughly characterize the livers into two groups after WD treatment; specifically, the Cisd2hKO^+/−^ mice were able to be separated from the WT and Cisd2TG mice (Figure 3A). Transcriptomic analysis revealed a list of 303 differentially expressed genes (239 upregulated genes and 64 downregulated genes) when the WD-fed WT and WD-fed Cisd2hKO^+/−^ mice were compared (Figure 3B,C). However, we were only able to detect five significantly upregulated genes (S100a1, Cisd2, Acpp, Ifih1 and Atpbd4) and no significantly downregulated genes, when the WD-fed Cisd2TG and WD-fed WT mice were compared (Figure 3B). Gene-annotation analysis was carried out to pinpoint the enriched biological processes associated with the genes that are differentially expressed in the Cisd2hKO^+/−^ mice. The findings suggest that the major significant changes that occurred were related to inflammation (leukocyte chemotaxis), lipid metabolism (sterol biosynthetic process) and DNA replication (DNA biosynthetic process and repair, etc.) (Figure 3D). Importantly, all of these changes in biological processes are consistent with the pathological and biochemical analysis findings.

### 3.3. The Differentially Expressed Genes that Are Affected by Cisd2 Haploinsufficiency

**Figure 4:**
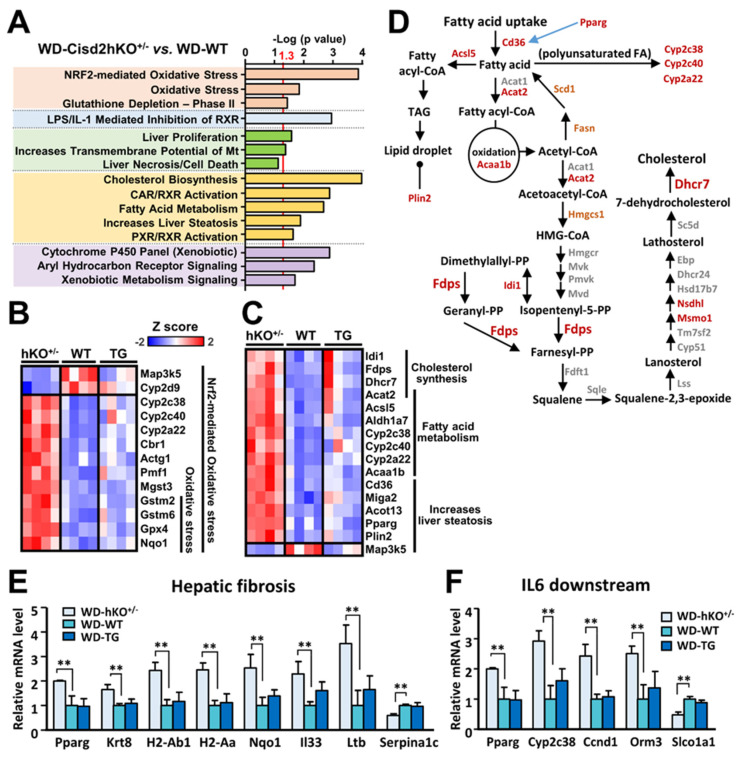
Functional analyses of differentially expressed genes in the livers of WD-fed mice. (**A**) Top lists from IPA using hepatic mRNA transcripts affected by hepatocyte-specific Cisd2-haploinsufficiency (Cisd2hKO^+/−^). (**B**,**C**) Heat maps of changes in the mRNA levels of genes related to oxidative stress (**B**) and lipid metabolism (**C**). (**D**) Cisd2hKO^+/−^ mice fed Western diet display altered expression of genes related to cholesterol biosynthesis. The significant genes (FDR < 0.05) are highlighted with red color. Borderline significance (FDR ≤ 0.1) of Hmgcs1, Fasn, and Scd1 are highlighted with orange color. (**E**) Expression levels of fibrosis-related genes from Tox list of ‘Hepatic Fibrosis’ in IPA (*n* = 4). (**F**) Expression levels of the downstream genes of IL-6 that is predicted to be activated in Cisd2hKO^+/−^ mice by IPA upstream regulator analysis *(n* = 4). WT control group was set as 1. Data are presented as mean ± SD. * *p* < 0.05; ** *p* < 0.005.

Using IPA software, Nrf2-mediated oxidative stress, LPS/IL-1-mediated pathway, cholesterol biosynthesis, and fatty acid metabolism were found to be among the top functional processes and/or pathways annotated by IPA (Figure 4A). Interestingly, the most significant observation is that the changes in mRNA expression affected by Cisd2 haploinsufficiency are ones that involve the induction of genes involved in Nrf2-mediated oxidative stress, as well as those involved in cholesterol biosynthesis and fatty acid metabolism (Figure 4B,C). In the liver, cholesterol can be derived from either the animal’s diet or by de novo synthesis via the mevalonate pathway. Notably, two critical enzymes in the latter pathway were found to be induced in WD-fed Cisd2hKO^+/−^ mice, namely farnesyl diphosphate synthase (Fdps) and 7-dehydrocholesterol reductase (Dhcr7). An overview of the interconnection between fatty acid metabolism and the cholesterol synthesis pathway is presented in Figure 4D. Moreover, in the WD-fed Cisd2hKO^+/−^ mice, despite the fact that the “hepatic fibrosis” pathway and the “IL-6-mediated” pathway are not selected as within the top list annotated by IPA, a certain number of genes involved in these two pathways are also significantly induced in the WD-fed Cisd2hKO^+/−^ mouse livers compared with those of the WT mice. In the “hepatic fibrosis” pathway, eight fibrogenic genes (Pparg, Krt8, H2-Ab1, H2-Aa, Nqo1, Il33, and Ltb) are upregulated, and one gene (Serpina1c) is downregulated (Figure 4E). In the “IL-6-mediated” pathway, four IL-6 downstream genes (Pparg, Cyp2c38, Ccnd1, and Orm3) are upregulated, and one gene (Slco1a1) is downregulated (Figure 4F). These findings are consistent with the pathological findings (Figure 2A), namely that severe inflammation occurs in the livers of WD-treated Cisd2hKO^+/−^ mice together with fibrosis.

### 3.4. Cisd2 Protects the Liver from Oxidative Stress and Reduce the Presence of Deletions Affecting Mitochondrial DNA

**Figure 5:**
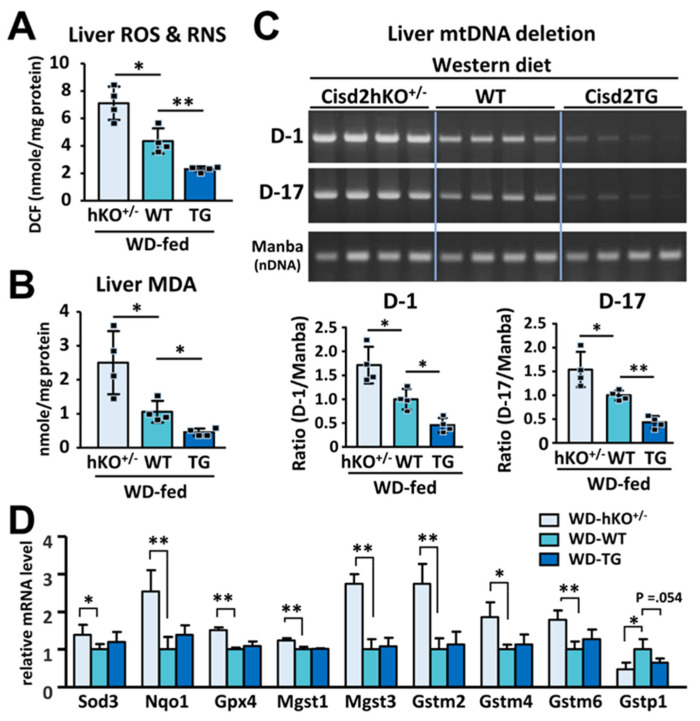
An inverse correlation between Cisd2 level and WD-induced oxidative stress in the liver. (**A**) Total reactive species levels (ROS/RNS) in the liver tissues of WD-fed mice (*n* = 4). (**B**) Hepatic contents of malondialdehyde (MDA) (*n* = 4). (**C**) Deletions of mitochondrial genome (mtDNA) in the liver tissues of WD-fed mice. Manba is a control nuclear gene (*n* = 4). (**D**) Differentially expressed genes involved in antioxidant defense in the liver tissues of WD-fed mice. The mean value of WT control group was set as 1 (*n* = 4). Data are presented as mean ± SD. * *p* < 0.05; ** *p* < 0.005.

ROS plays a crucial role in the progression of NAFLD toward NASH. In addition, ROS and abnormal lipid metabolism usually form a vicious cycle that exacerbates liver pathogenesis. Intriguingly, our results reveal a reverse correlation between the level of Cisd2 protein expression and oxidative stress, as indicated by both the relative level of total ROS/RNS (Figure 5A) and the concentration of malondialdehyde (MDA), which is a lipid oxidation product found in the liver (Figure 5B). Specifically, the Cisd2hKO^+/−^ mice (one copy of Cisd2) have a higher level of oxidative stress compared with the WT mice (two copies of Cisd2). On the other hand, the Cisd2TG mice (four copies of Cisd2) have lower oxidative stress compared with the WT mice. Furthermore, we are also able to provide further evidence to substantiate the hypothesis that Cisd2 is able to protect the liver from oxidative stress by investigating the presence of deleterious alterations to mitochondrial DNA (mtDNA) that happen after WD treatment. In the WD-fed Cisd2hKO^+/−^ mice, there is a significant increase of mtDNA deletions in the mitochondria from their livers compared with that the situation in the WD-fed WT mice. However, in the WD-fed Cisd2TG mice, which have four copies of Cisd2, there was a significant reduction of mtDNA deletions within their livers (Figure 5C).

Moreover, in the WD-fed Cisd2hKO^+/−^ mice, there are eight protective antioxidant enzymes upregulated in their livers, namely Sod3, Nqo1, Gpx4, Mgst1, Mgst3, Gstm2, Gstm4, and Gstm6. This is likely to be a compensatory effect occurring in response to the tremendous increase in oxidative stress in the livers of these mice. On the other hand, one antioxidant enzyme, Gstp1, is downregulated in the livers of WD-fed Cisd2hKO^+/−^ mice (Figure 5D). However, we found that there was no difference in the mRNA levels of these antioxidant genes when Cisd2TG and WT mice were compared. This suggests that there may be additional mechanisms whereby Cisd2 regulates redox homeostasis in the context of WD-induced metabolic dysregulation. Alternatively, this also could be attributable to the limited time that the WD was fed to the mice; that is for 4 months; thus, the WD treatment might be insufficient to induce a dramatic systematic change in terms of pathology (Figure 2) and global alterations to gene expression (Figure 3) between Cisd2TG and WT mice. Taken together, these findings suggest that Cisd2 haploinsufficiency predisposes mice to the development of NAFLD and increases their sensitivity to oxidative damage. Conversely, Cisd2 overexpression is able to effectively suppress the level of oxidative stress present in the mice fed a WD, thereby limiting lipotoxicity and mitochondrial DNA deletion, as well as attenuating the pathological damage brought about by chronic WD consumption.

## 4. Discussion

Here, we provide evidence to substantiate the hypothesis that Cisd2 plays a crucial role in protecting the liver from WD-induced detrimental effects and associated pathogenesis. Several discoveries can be pinpointed. Firstly, changes in the gene dosage of Cisd2 modulates the severity of WD-induced NAFLD. Specifically, Cisd2 haploinsufficiency, namely one copy of Cisd2 in the liver, is insufficient and this accelerates the pathogenesis of NAFLD and exacerbates progression toward NASH. Conversely, an enhanced level of Cisd2 is able to attenuate WD-induced NAFLD, thereby preventing the development of NASH at a later stage. Secondly, consistent with our pathological findings, transcriptomics analysis reveals that the most significant changes in biological processes/pathways are related to inflammation, lipid metabolism, and DNA replication/repair. Thirdly, among the differentially expressed genes affected by Cisd2 haploinsufficiency, the most significant observation is that there are changes in mRNA expression levels involving the induction of genes involved in Nrf2-mediated oxidative stress, as well as in cholesterol biosynthesis and fatty acid metabolism. Finally, Cisd2 protein expression seems to be able to protect the liver from oxidative stress and reduces the presence of mitochondrial DNA deletions.

### 4.1. The Nrf2-Mediated Oxidative Stress Pathway in NAFLD and NASH

In this study, our findings reveal a negative correlation in the liver between the level of Cisd2 protein expression and the degree of oxidative injury and liver pathology. Our research also has elucidated the mechanism underlying the protective effects of Cisd2 regarding the development of NAFLD and NASH. In the livers of WD-fed Cisd2hKO^+/−^ mice, transcriptome analysis shows an overt induction of the genes involved in steatosis, inflammation, DNA replication/repair, and the Nrf2 pathway. In contrast, in the livers of WD-fed Cisd2TG mice, the absence of or only minimal changes in the transcriptome of these mouse livers were observed relative to WD-fed WT mice (Figure 4). Nuclear factor E2-related factor 2 (Nrf2) is one of the most important transcription factors that brings about protection against hyperoxic injury, and it does this by inducing various cytoprotective enzymes [30]. For example, one of Nrf2’s downstream targets is NADPH Quinone oxidoreductase (NQO1), which protects against diet-induced metabolic defects [31]. In fact, Nrf2 knockout mice have been shown to be more susceptible to high-fat diet-induced hepatic steatosis [32]. Therefore, activation of the Nrf2 pathway seems to be a compensatory mechanism the occurs in response to the oxidative stress induced by a high-fat diet or a Western diet. Based on the above findings, the relationship between Cisd2 and Nrf2 is deserving of further investigation, and such investigations should be able to reveal the possible mechanism(s) underlying Cisd2-mediated protection of the liver.

### 4.2. Cholesterol Biosynthesis and Fatty Acid Metabolism in WD-Induced NAFLD

Under current WD dietary regimes, cholesterol biosynthesis is one of the most significantly altered metabolic pathways in the livers of Cisd2hKO^+/−^ mice, as indicated by the elevation of a panel of genes involved in the mevalonate pathway (Figure 3D and Figure 4D). In addition, in the WD-fed Cisd2hKO^+/−^ mice, increased mRNA levels of peroxisome proliferator-activated receptor gamma (PPARγ), long-chain acyl-CoA synthetase 5 (Acsl5), lipid droplet coat protein perilipin 2 (Plin2), and fatty acid transporter (Cd36) are also detectable in the liver; this suggests that Cisd2 may also modulate gene networks that promote lipid synthesis and storage in either a direct or indirect manner. By way of contrast, we did not observe any upregulation of genes related to fatty acid oxidation except for peroxisomal 3-ketoacyl-CoA thiolase (Acaa1b) (Figure 4C,D). Collectively, three dysregulated lipid metabolism processes, namely increased lipogenic capacity, decreased lipid catabolism, and elevated cholesterol synthesis, are all likely to contribute to the accumulation of fat in the liver, which is followed by a rapid progression from NAFLD to NASH when there is Cisd2 haploinsufficiency.

### 4.3. Clinical Implications: Activation of CISD2 Is a Promising Therapeutic Strategy for the Treatment of NAFLD and NASH

Despite the high prevalence of NAFLD/NASH and the high risk of serious progression to fibrosis and HCC, no drugs that specifically target NAFLD or NASH have been approved [3]. Currently, potential therapeutic strategies include lifestyle interventions (i.e., exercise and diet restriction), surgical approaches (bariatric surgery) and administration of antioxidants [33]. Interestingly, some interventions seem to be associated with enhanced Cisd2 expression in mammals. For example, one of our recent studies has demonstrated that treadmill exercise for 56 days is able to significantly enhances Cisd2 expression using a Cisd2-luciferase reporter mouse model [34]. Moreover, voluntary exercise for four weeks is able to increase Cisd2 protein levels in the skeletal muscle and white adipose tissue of mice [35]. Furthermore, in humans, CISD2 protein has been found to be upregulated in the skeletal muscle of obese patients after Roux-en-Y gastric bypass surgery [36]. Additionally, several studies have revealed that certain natural antioxidant treatments, such as curcumin and bitter melon extract, are able to ameliorate NAFLD in mice [37,38]. Interestingly, these two natural products have also been reported to increase Cisd2 levels in mice [39,40]. However, it is unclear whether the mechanism of action of these interventions is dependent on Cisd2. Thus, there is clearly a need for further investigations in these areas.

### 4.4. CISD2 May Function as a Double-Edged Sword during Cancer Development

Intriguingly, in contrast to the tumor-suppressive role of Cisd2 at an early stage during spontaneous development of HCC in a mouse model [20], it seems that human CISD2 protein is associated with oncogenic properties after malignant transformation in various cancer cell types, including HCC [41]. In normal hepatocytes, CISD2 is essential to maintaining Ca^2+^ and redox homeostasis, and to ameliorating cellular damages thereby pre-venting the development of NASH, which is a promoting factor of HCC, in the liver. On the other hand, in HCC cancer cells, CISD2 may also benefit tumor cells by supporting energetic and metabolic demands via mediating mitochondrial functions. Accordingly, the paradoxical role of CISD2, namely oncogenic or tumor-suppressive, is dependent on the stage during tumor development [13]. In this context, targeted inhibition of CISD2 specifically in the cancer cells rather than CISD2 activation may be more appreciated to benefit the HCC patients.

## 5. Conclusions

Cisd2 is a molecular target for the treatment of NAFLD and NASH. NAFLD, and its more severe form NASH, are the most common chronic human liver diseases. They are also among the main risk factors for HCC. Development of therapeutic agents that are able to bring about an effective enhancement of expression of the Cisd2 might be a potentially therapeutic strategy for the treatment of fatty liver diseases induced by a Western diet. The upregulation of Cisd2 expression by a Cisd2 activator should be able to ameliorate intracellular oxidative stress, help maintain metabolic homeostasis, and bring about a reversal of steatosis, thereby preventing a subsequent malignant progression to cirrhosis and HCC.

## Data Availability

Not applicable.

## References

[1] Cotter T.G., Rinella M. (2020). Nonalcoholic fatty liver disease 2020: The state of the disease. Gastroenterology.

[2] Anstee Q.M., Reeves H.L., Kotsiliti E., Govaere O., Heikenwalder M. (2019). From NASH to HCC: Current concepts and future challenges. Nat. Rev. Gastroenterol. Hepatol..

[3] Marjot T., Moolla A., Cobbold J.F., Hodson L., Tomlinson J.W. (2020). Nonalcoholic fatty liver disease in adults: Current concepts in etiology, outcomes, and management. Endocr. Rev..

[4] Tilg H., Moschen A.R. (2010). Evolution of inflammation in nonalcoholic fatty liver disease: The multiple parallel hits hypothesis. Hepatology.

[5] Chen Z., Tian R., She Z., Cai J., Li H. (2020). Role of oxidative stress in the pathogenesis of nonalcoholic fatty liver disease. Free Radic. Biol. Med..

[6] Neuschwander-Tetri B.A. (2010). Hepatic lipotoxicity and the pathogenesis of nonalcoholic steatohepatitis: The central role of nontriglyceride fatty acid metabolites. Hepatology.

[7] Masarone M., Rosato V., Dallio M., Gravina A.G., Aglitti A., Loguercio C., Federico A., Persico M. (2018). Role of oxidative stress in pathophysiology of nonalcoholic fatty liver disease. Oxid. Med. Cell. Longev..

[8] Chen Y.F., Kao C.H., Chen Y.T., Wang C.H., Wu C.Y., Tsai C.Y., Liu F.C., Yang C.W., Wei Y.H., Hsu M.T. (2009). Cisd2 deficiency drives premature aging and causes mitochondria-mediated defects in mice. Genes Dev..

[9] Chen Y.F., Wu C.Y., Kirby R., Kao C.H., Tsai T.F. (2010). A role for the CISD2 gene in lifespan control and human disease. Ann. N. Y. Acad. Sci..

[10] Wu C.Y., Chen Y.F., Wang C.H., Kao C.H., Zhuang H.W., Chen C.C., Chen L.K., Kirby R., Wei Y.H., Tsai S.F. (2012). A persistent level of Cisd2 extends healthy lifespan and delays aging in mice. Hum. Mol. Genet..

[11] Yeh C.H., Shen Z.Q., Hsiung S.Y., Wu P.C., Teng Y.C., Chou Y.J., Fang S.W., Chen C.F., Yan Y.T., Kao L.S. (2019). Cisd2 is essential to delaying cardiac aging and to maintaining heart functions. PLoS Biol..

[12] Yeh C.H., Chou Y.J., Kao C.H., Tsai T.F. (2020). Mitochondria and Calcium Homeostasis: Cisd2 as a big player in cardiac ageing. Int. J. Mol. Sci..

[13] Shen Z.Q., Huang Y.L., Teng Y.C., Wang T.W., Kao C.H., Yeh C.H., Tsai T.F. (2021). CISD2 maintains cellular homeostasis. Biochim. Biophys. Acta Mol. Cell Res..

[14] Chang N.C., Nguyen M., Bourdon J., Risse P.A., Martin J., Danialou G., Rizzuto R., Petrof B.J., Shore G.C. (2012). Bcl-2-associated autophagy regulator Naf-1 required for maintenance of skeletal muscle. Hum. Mol. Genet..

[15] Wiley S.E., Andreyev A.Y., Divakaruni A.S., Karisch R., Perkins G., Wall E.A., van der Geer P., Chen Y.F., Tsai T.F., Simon M.I. (2013). Wolfram Syndrome protein, Miner1, regulates sulphydryl redox status, the unfolded protein response, and Ca^2+^ homeostasis. EMBO Mol. Med..

[16] Lu S., Kanekura K., Hara T., Mahadevan J., Spears L.D., Oslowski C.M., Martinez R., Yamazaki-Inoue M., Toyoda M., Neilson A. (2014). A calcium-dependent protease as a potential therapeutic target for Wolfram syndrome. Proc. Natl. Acad. Sci. USA.

[17] Wang C.H., Chen Y.F., Wu C.Y., Wu P.C., Huang Y.L., Kao C.H., Lin C.H., Kao L.S., Tsai T.F., Wei Y.H. (2014). Cisd2 modulates the differentiation and functioning of adipocytes by regulating intracellular Ca^2+^ homeostasis. Hum. Mol. Genet..

[18] Wang C.H., Kao C.H., Chen Y.F., Wei Y.H., Tsai T.F. (2014). Cisd2 mediates lifespan: Is there an interconnection among Ca^2+^ homeostasis, autophagy, and lifespan?. Free Radic. Res..

[19] Wang C.H., Tsai T.F., Wei Y.H. (2015). Role of mitochondrial dysfunction and dysregulation of Ca^2+^ homeostasis in insulin insensitivity of mammalian cells. Ann. N. Y. Acad. Sci..

[20] Shen Z.Q., Chen Y.F., Chen J.R., Jou Y.S., Wu P.C., Kao C.H., Wang C.H., Huang Y.L., Chen C.F., Huang T.S. (2017). CISD2 haploinsufficiency disrupts calcium homeostasis, causes nonalcoholic fatty liver disease, and promotes hepatocellular carcinoma. Cell Rep..

[21] Shen Z.Q., Huang Y.L., Tsai T.F. (2018). Cisd2 haploinsufficiency: A driving force for hepatocellular carcinoma. Mol. Cell. Oncol..

[22] Chakravarthy M.V., Neuschwander-Tetri B.A. (2020). The metabolic basis of nonalcoholic steatohepatitis. Endocrinol. Diabetes Metab..

[23] Charlton M., Krishnan A., Viker K., Sanderson S., Cazanave S., McConico A., Masuoko H., Gores G. (2011). Fast food diet mouse: Novel small animal model of NASH with ballooning, progressive fibrosis, and high physiological fidelity to the human condition. Am. J. Physiol. Gastrointest. Liver Physiol..

[24] Peng C., Stewart A.G., Woodman O.L., Ritchie R.H., Qin C.X. (2020). Non-alcoholic steatohepatitis: A review of its mechanism, models and medical treatments. Front. Pharmacol..

[25] Tsuchida T., Lee Y.A., Fujiwara N., Ybanez M., Allen B., Martins S., Fiel M.I., Goossens N., Chou H.I., Hoshida Y. (2018). A simple diet- and chemical-induced murine NASH model with rapid progression of steatohepatitis, fibrosis and liver cancer. J. Hepatol..

[26] Bligh E.G., Dyer W.J. (1959). A rapid method of total lipid extraction and purification. Can. J. Biochem. Physiol..

[27] Tanhauser S.M., Laipis P.J. (1995). Multiple deletions are detectable in mitochondrial DNA of aging mice. J. Biol. Chem..

[28] Saeed A.I., Sharov V., White J., Li J., Liang W., Bhagabati N., Braisted J., Klapa M., Currier T., Thiagarajan M. (2003). TM4: A free, open-source system for microarray data management and analysis. BioTechniques.

[29] Korinkova L., Prazienkova V., Cerna L., Karnosova A., Zelezna B., Kunes J., Maletinska L. (2020). Pathophysiology of NAFLD and NASH in experimental models: The role of food intake regulating peptides. Front. Endocrinol..

[30] Schmidlin C.J., Dodson M.B., Madhavan L., Zhang D.D. (2019). Redox regulation by NRF2 in aging and disease. Free Radic. Biol. Med..

[31] Di Francesco A., Choi Y., Bernier M., Zhang Y., Diaz-Ruiz A., Aon M.A., Kalafut K., Ehrlich M.R., Murt K., Ali A. (2020). NQO1 protects obese mice through improvements in glucose and lipid metabolism. NPJ Aging Mech. Dis..

[32] Meakin P.J., Chowdhry S., Sharma R.S., Ashford F.B., Walsh S.V., McCrimmon R.J., Dinkova-Kostova A.T., Dillon J.F., Hayes J.D., Ashford M.L. (2014). Susceptibility of Nrf2-null mice to steatohepatitis and cirrhosis upon consumption of a high-fat diet is associated with oxidative stress, perturbation of the unfolded protein response, and disturbance in the expression of metabolic enzymes but not with insulin resistance. Mol. Cell. Biol..

[33] Raza S., Rajak S., Upadhyay A., Tewari A., Anthony Sinha R. (2021). Current treatment paradigms and emerging therapies for NAFLD/NASH. Front. Biosci..

[34] Teng Y.C., Wang J.Y., Chi Y.H., Tsai T.F. (2020). Exercise and the Cisd2 prolongevity gene: Two promising strategies to delay the aging of skeletal muscle. Int. J. Mol. Sci..

[35] Yokokawa T., Kido K., Suga T., Sase K., Isaka T., Hayashi T., Fujita S. (2018). Exercise training increases CISD family protein expression in murine skeletal muscle and white adipose tissue. Biochem. Biophys. Res. Commun..

[36] Campbell L.E., Langlais P.R., Day S.E., Coletta R.L., Benjamin T.R., De Filippis E.A., Madura J.A., Mandarino L.J., Roust L.R., Coletta D.K. (2016). Identification of novel changes in human skeletal muscle proteome after roux-en-y gastric bypass surgery. Diabetes.

[37] Lee D.E., Lee S.J., Kim S.J., Lee H.S., Kwon O.S. (2019). Curcumin ameliorates nonalcoholic fatty liver disease through inhibition of O-glcnacylation. Nutrients.

[38] Dwijayanti D.R., Shimada T., Ishii T., Okuyama T., Ikeya Y., Mukai E., Nishizawa M. (2020). Bitter melon fruit extract has a hypoglycemic effect and reduces hepatic lipid accumulation in ob/ob mice. Phytother. Res. PTR.

[39] Kung W.M., Lin C.C., Kuo C.Y., Juin Y.C., Wu P.C., Lin M.S. (2020). Wild bitter melon exerts anti-inflammatory effects by upregulating injury-attenuated CISD2 expression following spinal cord injury. Behav. Neurol..

[40] Lin C.C., Chiang T.H., Sun Y.Y., Lin M.S. (2019). Protective Effects of CISD2 and influence of curcumin on CISD2 expression in aged animals and inflammatory cell model. Nutrients.

[41] Chen B., Shen S., Wu J., Hua Y., Kuang M., Li S., Peng B. (2015). CISD2 associated with proliferation indicates negative prognosis in patients with hepatocellular carcinoma. Int. J. Clin. Exp. Pathol..

